# Epidemiology of breast cancer subtypes in two prospective cohort studies of breast cancer survivors

**DOI:** 10.1186/bcr2261

**Published:** 2009-05-22

**Authors:** Marilyn L Kwan, Lawrence H Kushi, Erin Weltzien, Benjamin Maring, Susan E Kutner, Regan S Fulton, Marion M Lee, Christine B Ambrosone, Bette J Caan

**Affiliations:** 1 Kaiser Permanente, Division of Research, 2000 Broadway, First Floor, Oakland, CA 94612, USA; 2 Kaiser Permanente, San Jose, 280 Hospital Parkway, San Jose, CA 95119, USA; 3 Kaiser Permanente, Regional Immunohistochemistry Laboratory, 350 St. Joseph's Street, San Francisco, CA 94115, USA; 4 Department of Epidemiology and Biostatistics, Box 0981, 185 Berry Street 6600, University of California, San Francisco, CA 94143-0981, USA; 5 Department of Cancer Prevention and Control, Roswell Park Cancer Institute, Elm & Carlton Streets, Buffalo, NY 14263, USA

## Abstract

**Introduction:**

The aim of this study was to describe breast tumor subtypes by common breast cancer risk factors and to determine correlates of subtypes using baseline data from two pooled prospective breast cancer studies within a large health maintenance organization.

**Methods:**

Tumor data on 2544 invasive breast cancer cases subtyped by estrogen receptor, progesterone receptor, and human epidermal growth factor receptor 2 (Her2) status were obtained (1868 luminal A tumors, 294 luminal B tumors, 288 triple-negative tumors and 94 Her2-overexpressing tumors). Demographic, reproductive and lifestyle information was collected either in person or by mailed questionnaires. Case-only odds ratios (ORs) and 95% confidence intervals (CIs) were estimated using logistic regression, adjusting for age at diagnosis, race/ethnicity, and study origin.

**Results:**

Compared with luminal A cases, luminal B cases were more likely to be younger at diagnosis (*P *= 0.0001) and were less likely to consume alcohol (OR = 0.74, 95% CI = 0.56 to 0.98), use hormone replacement therapy (HRT) (OR = 0.66, 95% CI = 0.46 to 0.94), and oral contraceptives (OR = 0.73, 95% CI = 0.55 to 0.96). Compared with luminal A cases, triple-negative cases tended to be younger at diagnosis (*P *≤ 0.0001) and African American (OR = 3.14, 95% CI = 2.12 to 4.16), were more likely to have not breastfed if they had parity greater than or equal to three (OR = 1.68, 95% CI = 1.00 to 2.81), and were more likely to be overweight (OR = 1.82, 95% CI = 1.03 to 3.24) or obese (OR = 1.97, 95% CI = 1.03 to 3.77) if premenopausal. Her2-overexpressing cases were more likely to be younger at diagnosis (*P *= 0.03) and Hispanic (OR = 2.19, 95% CI = 1.16 to 4.13) or Asian (OR = 2.02, 95% CI = 1.05 to 3.88), and less likely to use HRT (OR = 0.45, 95% CI = 0.26 to 0.79).

**Conclusions:**

These observations suggest that investigators should consider tumor heterogeneity in associations with traditional breast cancer risk factors. Important modifiable lifestyle factors that may be related to the development of a specific tumor subtype, but not all subtypes, include obesity, breastfeeding, and alcohol consumption. Future work that will further categorize triple-negative cases into basal and non-basal tumors may help to elucidate these associations further.

## Introduction

Among women in the USA, breast cancer remains the most commonly diagnosed cancer, excluding skin cancers, and the second leading cause of cancer-related death [1]. Breast cancer is characterized by its molecular and clinical heterogeneity. Studies using cDNA microarrays and immunohistochemical (IHC) markers [2-6] have classified breast cancers into five distinct subtypes: luminal A (estrogen receptor (ER) positive and/or progesterone receptor (PR) positive, human epidermal growth factor receptor 2 (Her2) negative), luminal B (ER positive and/or PR positive, Her2 positive), Her2 overexpressing (ER negative, PR negative, Her2 positive), basal-like (ER negative, PR negative, Her2 negative, cytokeratin (CK) 5/6 positive and/or epidermal growth factor receptor (EGFR) positive) and normal breast-like tumors. Approximately 70% of 'triple-negative' breast cancers (ER negative, PR negative, Her2 negative) express basal markers [7-9], resulting in the triple-negative subtype commonly being used as a surrogate marker for the basal-like subtype.

Luminal tumors have been associated with the most favorable prognoses, while Her2-overexpressing and basal-like tumors, or their surrogate triple negative tumors, have been associated with the worst prognoses [2,3,5,6,10-18]. For triple-negative tumors, the peak risk of recurrence occurs within three years of diagnosis, and mortality rates are increased for five years after diagnosis [14,19]. The subtype accounts for approximately 15% of invasive breast cancers [2,4,10,11,20] and is commonly associated with African American race [2,10,17,20-22], younger age at diagnosis [10,11,14,16,17,19,20,22-24], more advanced stage [10,11,24], higher grade [2,5,9,11,14,16,17,19,21,24,25], high mitotic indices [2,16,26], family history of breast cancer [27], and BRCA1 mutations [23,28,29].

Although many studies have examined associations between common breast cancer risk factors, race [30-36] and hormone receptor status [36-41], few studies have explored the relationship between common breast cancer risk factors and the molecular subtypes of breast cancer [22,27,42,43] [see Additional data file 1]. Therefore, we set out to describe breast tumor subtypes by race/ethnicity and common breast cancer risk factors and to determine correlates of breast cancer subtypes using baseline data from two large, prospective breast cancer survivorship studies of 2544 invasive breast cancer cases.

## Materials and methods

### LACE Study

The Life After Cancer Epidemiology (LACE) Study consists of 2280 women diagnosed with invasive breast cancer between 1997 and 2000 and recruited primarily from the Kaiser Permanente Northern California (KPNC) Cancer Registry (82%) and the Utah Cancer Registry (12%). Further details on the LACE cohort have been previously reported [44]. Briefly, eligibility criteria included age between 18 and 70 years at enrollment; a diagnosis of early-stage primary breast cancer (stages I ≥ 1 cm, II, or IIIA); enrollment between 11 and 39 months post-diagnosis; having completed breast cancer treatment (except for adjuvant hormonal therapy); free of recurrence; and no history of other cancers in the five years prior to enrollment. Between January 2000 and April 2002, 2280 eligible women completed baseline questionnaires via mail. The mean time from diagnosis to enrollment was 22.8 months (range = 11.0 to 38.9 months). The study was approved by the institutional review boards (IRB) of KPNC and the University of Utah. The present analysis includes data from 1821 KPNC breast cancer patients from the LACE Study with complete breast cancer subtype information.

### Pathways Study

The Pathways Study is a prospective cohort study actively recruiting women diagnosed with invasive breast cancer from the KPNC patient population since January 2006. Women are recruited as soon after diagnosis as possible (usually within two months), as described elsewhere [45]. Briefly, cases are rapidly ascertained on a daily basis by automatic scanning of electronic pathology reports with subsequent verification of cancer diagnosis and patient notification by a medical record analyst. Eligibility criteria include: current KPNC membership; at least 21 years of age at diagnosis; recent diagnosis of first primary invasive breast cancer (all stages); no prior history of any cancer; ability to speak English, Spanish, Cantonese, or Mandarin; and live within a 65-mile radius of a field interviewer. In addition, a passive consent is obtained from the patient's physician of record by an email notification stating our intention to contact the patient for study recruitment. Recruitment is ongoing, and as of 20 October, 2008, 2212 breast cancer patients have been enrolled via in-person interview. The mean time from diagnosis to enrollment is 1.9 months (range = 0 to 7.3 months). Written informed consent is obtained from all participants before they are enrolled in the study, typically at the time of the in-person baseline interview. The study was approved by the IRB of KPNC and all collaborating sites. In order to make these cases comparable with those from the LACE Study, the present analysis includes data from the first 723 women enrolled with a diagnosis of stages I ≥ 1 cm, II, or IIIA breast cancer and having complete breast cancer subtype data.

### Data collection

#### Reproductive and lifestyle factors

In the mailed baseline questionnaire of the LACE Study and during the in-person baseline interview of the Pathways Study, participants were asked detailed information on family history of cancer and reproductive history, including age at first full-term pregnancy, number of biological children, breastfeeding, and menopausal status. Additional information was collected on smoking, alcohol use, hormone use (oral contraceptives (OC), hormone replacement therapy (HRT)), and demographics (age at breast cancer diagnosis, race/ethnicity, household income, education). Self-reported height and weight one year before diagnosis (LACE) and around diagnosis (Pathways) was obtained to calculate body mass index (BMI, kg/m^2^). Any missing values were supplemented by concurrent information from KPNC electronic medical records.

#### Tumor characteristics

Data on ER and PR status and Her2 expression were obtained from medical record review and the KPNC Cancer Registry [46] for LACE and from the KPNC Cancer Registry and other KPNC databases for Pathways. Data are collected, coded, and added to the KPNC Cancer Registry approximately four months after diagnosis to allow for the completion of treatment. For all breast surgical specimens, hormone receptor status, and Her2 expression is determined by IHC at the KPNC regional IHC laboratory and has been reported to the KPNC Cancer Registry since January 2000. Gene expression profiling studies have shown that IHC of paraffin sections is a reliable surrogate for molecular classification of invasive breast cancers [3,47-51]. Beginning in July 1999, if the IHC staining for Her2 expression is equivocal (less than 30% strong staining, but more than 10% weak staining), then the specimen is sent for fluorescence *in situ *hybridization (FISH) at the KPNC regional cytogenetics laboratory. If the FISH score (Her2: 17 cen) is less than 2.0 [52], then the woman is classified as having Her2-negative tumor expression; if the FISH score is greater than 2.0, then the woman is classified as having Her2-positive tumor expression. Results from FISH analyses are not reported to the KPNC Cancer Registry, and are obtained directly from the KPNC regional cytogenetics laboratory.

### Covariate classification

Demographic, reproductive, and lifestyle covariates of interest were classified as follows: age at diagnosis (< 50, 50 to 64, ≥ 65 years), race/ethnicity (white, African American, Hispanic, Asian, other), menopausal status (pre, post), family history of breast cancer (no, yes), parity (nulliparous, 1 to 2, ≥ 3 children), age at first full-term pregnancy (nulliparous, < 26, ≥ 26 years), lifetime duration of lactation (never, 0 to 3, ≥ 4 months), alcohol use (never, ever), smoking duration (never, ≤ 10, 11 to 19, ≥ 20 years), hormone replacement therapy (HRT) among post-menopausal women (never, ever), OC use (never, ever), and BMI (< 25, 25 to 29, ≥ 30 kg/m^2^).

### Outcome classification

Although the presence of basal markers can significantly improve the prognostic value of the triple-negative phenotype [13], for this analysis, we did not have IHC data for CK5/6 and EGFR expression. Thus, we were unable to further classify triple-negative cases into basal-like and non-basal-like breast tumors. Considering this limitation, the tumor subtype groups in this analysis consisted of: ER positive and/or PR positive, and Her2 negative (luminal A); ER positive and/or PR positive, and Her2 positive (luminal B); ER negative, PR negative, and Her2 negative (triple negative); ER negative, PR negative, and Her2 positive (Her2-overexpressing).

### Statistical analysis

Comparisons of demographic, reproductive, and lifestyle characteristics by cohort study and race/ethnicity were conducted using Pearson chi-square tests. Using the combined sample size of 2544 breast cancer survivors, case-only odds ratios (ORs) and 95% confidence intervals (CIs) were estimated using logistic regression. The luminal A group was selected as the referent because the majority of invasive breast cancer cases are of this subtype. All models were adjusted for age at diagnosis, race/ethnicity, and Pathways/LACE study origin except when these covariates were the predictors of interest. We also examined whether the associations between parity and tumor subtype varied by breastfeeding and whether BMI and tumor subtype varied by menopausal status by first generating strata-specific estimates and then including an interaction term in the model to test for statistical significance. CIs not overlapping with 1.00 or *P *< 0.05 were considered statistically significant.

## Results

Demographic, reproductive, and lifestyle factors varied significantly by race/ethnicity in the combined studies (Table 1). Demographically, African Americans (mean age = 56.2 years) and Asians (mean age = 54.8 years) were more likely to be diagnosed at a younger age although whites were more likely to be diagnosed at an older age (mean age = 59.8 years). However, Asians (59.1%) were less likely to be post-menopausal than whites (75.6%), African Americans (71.4%), and other races/ethnicities (72.9%). A positive family history of breast cancer was more common among whites (22.6%) and other races/ethnicities (24.1%), than among the other groups. Reproductive history also differed markedly by race/ethnicity; African Americans and Hispanics had more biological children (42.6% and 44.2%, respectively) and were younger during their first pregnancy (73.4% and 60.7%) compared with the other races/ethnicities. Whites were more likely to have ever consumed alcohol (60.1%) while Asians (78.3%) were more likely to have never smoked. Among the hormonal factors, more whites had used HRT (76.2%) compared with the other races/ethnicities, while fewer Asians (44.1%) had used OCs compared with the other races/ethnicities. African Americans were more obese at diagnosis (49.0%) followed by other races/ethnicities (37.0%) and Hispanics (34.5%). Additional data file 2 shows the distribution of demographic, reproductive, and lifestyle factors in the LACE and Pathways Studies separately. Overall, the two study populations were similar demographically, yet non-similarities were apparent among reproductive and hormonal factors, likely to be due to differences in time periods of data collection.

**Table 1:** Distribution of demographic, reproductive, and lifestyle risk factors by race/ethnicity in the combined LACE and Pathways Studies (n = 2544)

	**White Total n = 1943 n (%)**	**African American Total n = 155 n (%)**	**Asian Total n = 189 n (%)**	**Hispanic Total n = 197 n (%)**	**Other Total n = 54 n (%)**	***P *value^a^**
Age at diagnosis (years)						< 0.0001
< 50	389 (20.0)	43 (27.7)	61 (32.3)	67 (34.0)	13 (24.1)	
50 to 64	879 (45.2)	80 (51.6)	87 (46.0)	80 (40.6)	29 (53.7)	
≥ 65	675 (34.7)	32 (20.6)	41 (21.7)	50 (25.4)	12 (22.2)	
mean (± standard deviation)	59.8 (11.2)	56.2 (10.7)	54.8 (11.0)	55.7 (12.4)	57.7 (9.8)	
Menopausal status						< 0.0001
Postmenopausal	1,330 (75.6)	90 (71.4)	104 (59.1)	117 (65.4)	35 (72.9)	
Premenopausal	430 (24.4)	36 (28.6)	72 (40.9)	62 (34.6)	13 (27.1)	
Family history						0.001
No	1,503 (77.4)	133 (85.8)	163 (86.2)	167 (84.8)	41 (75.9)	
Yes	440 (22.6)	22 (14.2)	26 (13.8)	30 (15.2)	13 (24.1)	
Parity						0.006
Nulliparous	343 (17.7)	22 (14.2)	46 (24.3)	31 (15.7)	9 (16.7)	
1 to 2 children	894 (46.0)	67 (43.2)	93 (49.2)	79 (40.1)	31 (57.4)	
≥ 3 children	706 (36.3)	66 (42.6)	50 (26.5)	87 (44.2)	14 (25.9)	
Age at first full-term pregnancy (years)						< 0.0001
Nulliparous	343 (17.7)	22 (14.3)	46 (24.3)	31 (15.8)	9 (16.7)	
< 26	1,020 (52.6)	113 (73.4)	61 (32.3)	119 (60.7)	28 (51.9)	
≥ 26	578 (29.8)	19 (12.3)	82 (43.4)	46 (23.5)	17 (31.5)	
Lifetime duration of lactation						0.215
Never	908 (47.3)	92 (59.7)	81 (44.3)	91 (48.1)	25 (47.2)	
0 to 3 months	279 (14.5)	19 (12.3)	31 (16.9)	25 (13.2)	8 (15.1)	
≥ 4 months	733 (38.2)	43 (27.9)	71 (38.8)	73 (38.6)	20 (37.7)	
Alcohol use						< 0.0001
Never	675 (39.9)	77 (67.5)	116 (75.3)	93 (62.4)	28 (57.1)	
Ever	1,016 (60.1)	37 (32.5)	38 (24.7)	56 (37.6)	21 (42.9)	
Smoking history (years)						< 0.0001
Never	925 (48.0)	73 (47.4)	148 (78.3)	113 (57.4)	22 (40.7)	
≤ 10	269 (14.0)	17 (11.0)	13 (6.9)	33 (16.8)	6 (11.1)	
11 to 19	169 (8.8)	10 (6.5)	8 (4.2)	6 (3.0)	5 (9.3)	
≥ 20	563 (29.2)	54 (35.1)	20 (10.6)	45 (22.8)	21 (38.9)	
Hormone replacement therapy (postmenopausal women only)						
Never	310 (23.8)	37 (42.1)	47 (45.6)	37 (32.2)	12 (34.3)	
Ever	994 (76.2)	51 (57.9)	56 (54.4)	78 (67.8)	23 (65.7)	
Oral contraceptive use						< 0.0001
Never	638 (33.8)	40 (26.7)	100 (55.9)	72 (37.7)	16 (30.2)	
Ever	1,252 (66.2)	110 (73.3)	79 (44.1)	119 (62.3)	37 (69.8)	
Body mass index (kg/m^2^)^b^						< 0.0001
< 25	835 (43.4)	31 (20.0)	119 (63.3)	63 (32.5)	20 (37.0)	
25 to 29	600 (31.1)	48 (31.0)	46 (24.5)	64 (33.0)	14 (25.9)	
≥ 30	491 (25.5)	76 (49.0)	23 (12.2)	67 (34.5)	20 (37.0)	

^a ^From Pearson chi-square test across racial/ethnic categories.

^b ^Body mass index one year pre-diagnosis (LACE) and around diagnosis (Pathways).

The distribution of breast cancer subtypes by race/ethnicity in the combined studies is presented in Table 2. Among the 2544 invasive breast cancer cases, 1868 (73.4%) were classified as luminal A, 294 (11.6%) as luminal B, 288 (11.3%) as triple negative, and 94 (3.7%) as Her2-overexpressing. The distribution of race/ethnicity within each subtype compared with all other subtypes varied significantly (*P *< 0.05). The majority of the whites (75.3%), Asians (71.4%), Hispanics (68.5%), other (68.5%), and African Americans (59.4%) had the luminal A tumor subtype. The Her2-overexpressing subtype was least common among all races/ethnicities (whites 3.1%, African Americans 3.2%, Asians 6.4%, Hispanics 6.6%, other 5.5%). African Americans had the highest prevalence of the triple negative subtype (28.4%) compared with the other races/ethnicities (whites 10.5%, Asians 6.3%, Hispanics 10.7%, other 13.0%).

**Table 2:** Distribution of breast cancer tumor subtypes by race/ethnicity in the combined LACE and Pathways Studies (n = 2544)

**Tumor subtype**	**White n (%)**	**African American n (%)**	**Asian n (%)**	**Hispanic n (%)**	**Other n (%)**	***P *value^a^**
Luminal A (ER+ and/or PR+, Her2-) n = 1868 (73.4)	1464 (75.3)	92 (59.4)	135 (71.4)	135 (68.5)	37 (68.5)	0.0001
Luminal B (ER+ and/or PR+, Her2+) n = 294 (11.6)	215 (11.1)	14 (9.0)	30 (15.9)	28 (14.2)	7 (13.0)	0.18
Triple negative (ER-, PR-, Her2-) n = 288 (11.3)	204 (10.5)	44 (28.4)	12 (6.3)	21 (10.7)	7 (13.0)	< 0.0001
Her2 overexpressing (ER-, PR-, Her2+) n = 94 (3.7)	60 (3.1)	5 (3.2)	12 (6.4)	13 (6.6)	3 (5.5)	0.03

Total n = 2544 (100)	1943 (100)	155 (100)	189 (100)	197 (100)	54 (100)	---

^a ^From Pearson chi-square test across racial/ethnic categories.

ER = estrogen receptor; Her2 = human epidermal growth receptor 2; PR = progesterone receptor; + = positive; - = negative.

The associations between various demographic, reproductive, and lifestyle factors within each subtype (luminal B, triple negative, and Her2-overexpressing) compared with luminal A are shown in Tables 3 and 4. All case-only ORs were adjusted for age, race/ethnicity, and Pathways/LACE study origin except when either age at diagnosis or race/ethnicity were the main predictors in the logistic regression model. Compared with luminal A cases, luminal B cases were more likely to be younger at diagnosis (OR for < 50 years = 1.83, 95% CI = 1.32 to 2.55; *P *= 0.0001) and were less likely to consume alcohol (OR = 0.74, 95% CI = 0.56 to 0.98), use HRT (OR = 0.66, 95% CI = 0.46 to 0.94), and OC (OR = 0.73, 95% CI = 0.55 to 0.96). Compared with luminal A cases, triple negative cases tended to be younger at diagnosis (OR for < 50 years = 2.78, 95% CI = 1.99 to 3.90; *P *≤ 0.0001) and African American (OR = 3.14, 95% CI = 2.12 to 4.16). Breastfeeding for at least four months was associated with being less likely to have a triple negative tumor, yet this association was of borderline significance (OR = 0.78, 95% CI = 0.59 to 1.03). Compared with luminal A cases, Her2-overexpressing cases were more likely to be younger at diagnosis (*P *= 0.03), similar to luminal B and triple negative cases, and less likely to use HRT (OR = 0.49, 95% CI = 0.26 to 0.79), similar to luminal B cases. Furthermore, Her2-overexpressing cases were more likely to be Hispanic (OR = 2.19, 95% CI = 1.16 to 4.13) and Asian (OR = 2.02, 95% CI = 1.05 to 3.88). The associations between the risk factors and tumor subtypes for the individual studies are presented in Additional data files 3 and 4. The majority of the case-only ORs were in the same direction as observed in the combined analysis, except for the association between BMI and the triple-negative subtype. In the LACE Study, triple-negative cases were more likely to have higher BMI while in the Pathways Study the opposite trend was observed, although these effect measures were not statistically significant.

**Table 3:** Case-only odds ratios and 95% confidence intervals from logistic regression models^a ^of associations between breast cancer tumor subtypes and demographic, reproductive, and lifestyle risk factors, combined LACE and Pathways Studies (n = 2544)

	**Luminal A (comparison)**	**Luminal B^a^**		
	**n**	**N**	**OR**	**95% CI**

Age at diagnosis (years)				
≥ 65 (Ref)	637	82	Ref	---
50 to 64	871	124	1.09	0.81, 1.47
< 50	355	88	1.83	1.32, 2.55
Test for trend				*P *= 0.0001
Race/ethnicity				
White (Ref)	1464	215	Ref	---
African American	92	14	0.97	0.54, 1.74
Hispanic	135	28	1.32	0.85, 2.04
Asian	135	30	1.38	0.90, 2.12
Other	37	7	1.29	0.56, 2.94
Menopausal status				---
Postmenopausal (Ref)	1283	173	Ref	
Premenopausal	406	95	1.32	0.87, 2.00
Family history				
No (Ref)	1453	245	Ref	---
Yes	410	49	0.74	0.53, 1.02
Age at first full-term pregnancy (years)				
Nulliparous (Ref)	335	51	Ref	---
< 26	959	158	1.25	0.88, 1.78
≥ 26	567	85	1.04	0.72, 1.52
Parity				
Nulliparous (Ref)	335	51	Ref	---
1 to 2 children	837	150	1.24	0.88, 1.76
≥ 3 children	691	93	1.03	0.70, 1.50
Lifetime duration of breastfeeding				
Never (Ref)	871	135	Ref	---
0 to 3 months	255	50	1.31	0.92, 1.88
≥ 4 months	711	100	0.86	0.65, 1.14
Parity among never breastfed^b^				
Nulliparous (Ref)	321	50	Ref	---
1 to 2 children	326	59	1.38	(0.89, 2.13)
≥ 3 children	^224^	26	1.01	(0.58, 1.76)
Parity among 0 to 3 months breastfed^b^				
1 to 2 children (Ref)	144	33	Ref	---
≥ 3 children	108	17	0.90	(0.45, 1.77)
Parity among ≥ 4 months breastfed^b^				
1 to 2 children (Ref)	358	56	Ref	---
≥ 3 children	352	44	0.84	(0.54, 1.32)
Alcohol use				
Never (Ref)	709	131	Ref	---
Ever	880	119	0.74	0.56, 0.98
Smoking history				
Never (Ref)	921	163	Ref	---
≤ 10	251	39	0.86	0.59, 1.27
11 to 19	147	22	0.93	0.57, 1.51
≥ 20	531	68	0.80	0.59, 1.09
Hormone replacement therapy (postmenopausal only)				
Never (Ref)	314	55	Ref	---
Ever	943	114	0.66	0.46, 0.94
Oral contraceptive use				
Never (Ref)	647	110	Ref	---
Ever	1160	172	0.73	0.55, 0.96
BMI (kg/m^2^)^c^				
< 25 (Ref)	785	134	Ref	---
25 to 29	563	79	0.92	0.67, 1.24
≥ 30	500	77	1.03	0.75, 1.41
BMI (kg/m^2^) among premenopausal^b^				
< 25 (Ref)	223	50	Ref	---
25 to 29	99	21	1.11	0.62, 2.00
≥ 30	81	24	1.68	0.92, 3.07
BMI (kg/m^2^) among postmenopausal^b^				
< 25 (Ref)	480	67	Ref	---
25 to 29	422	52	0.90	0.61, 1.33
≥ 30	372	50	0.99	0.66, 1.47

^a ^Adjusted for age at diagnosis, race/ethnicity, and Pathways/LACE study origin except in models with age at diagnosis or race/ethnicity as main predictors.

^b ^*P *for interaction not statistically significant (*P *> 0.05) in any tumor subtype model.

^c ^BMI = body mass index one year pre-diagnosis (LACE) and around diagnosis (Pathways).

CI = confidence interval; OR = odds ratio.

**Table 4:** Case-only odds ratios and 95% confidence intervals from logistic regression models^a ^of associations between breast cancer tumor subtypes and demographic, reproductive, and lifestyle risk factors, combined LACE and Pathways Studies (n = 2544)

	**Luminal A (comparison)**	**Triple negative^a^**			**HER2-overexpressing^a^**		
	**n**	**n**	**OR**	**95% CI**	**n**	**OR**	**95% CI**

Age at diagnosis (years)							
≥ 65 (Ref)	637	68	Ref	---	23	Ref	---
50 to 64	871	115	1.99	0.85, 1.62	45	1.39	0.83, 2.32
< 50	355	105	2.78	1.99, 3.90	25	1.75	0.97, 3.15
Test for trend				*P *≤ 0.0001			*P *= 0.03
Race/ethnicity							
White (Ref)	1464	204	Ref	---	60	Ref	---
African American	92	44	3.14	2.12, 4.66	5	1.25	0.49, 3.21
Hispanic	135	21	0.93	0.57, 1.53	13	2.19	1.16, 4.13
Asian	135	12	0.53	0.28, 0.97	12	2.02	1.05, 3.88
Other	37	7	1.28	0.56, 2.94	3	1.95	0.58, 6.52
Menopausal status							
Postmenopausal (Ref)	1283	163	Ref	---	57	Ref	---
Premenopausal	406	91	0.84	0.55, 1.27	21	0.65	0.31, 1.33
Family history							
No (Ref)	1453	229	Ref	---	80	Ref	---
Yes	410	59	0.95	0.69, 1.29	13	0.62	0.34, 1.13
Age at first full-term pregnancy (years)							
Nulliparous (Ref)	335	52	Ref	---	13	Ref	---
< 26	959	163	1.28	0.90, 1.82	61	2.02	1.07, 3.80
≥ 26	567	72	0.93	0.63, 1.38	18	0.86	0.42, 1.79
Parity							
Nulliparous (Ref)	335	52	Ref	---	13	Ref	---
1 to 2 children	837	137	1.11	0.78, 1.58	40	1.30	0.68, 2.47
≥ 3 children	691	99	1.18	0.81, 1.72	40	1.82	0.94, 3.53
Lifetime duration of breastfeeding							
Never (Ref)	871	148	Ref	---	43	Ref	---
0 to 3 months	255	41	1.04	0.71, 1.52	16	1.29	0.71, 2.35
≥ 4 months	711	97	0.78	0.59, 1.03	32	0.86	0.54, 1.38
Parity among never breastfed^b^							
Nulliparous (Ref)	321	50	Ref	---	12	Ref	---
1 to 2 children	326	59	1.34	(0.87, 2.08)	15	1.52	(0.68, 3.41)
≥ 3 children	^224^	39	1.68	(1.00, 2.81)	16	3.03	(1.27, 7.23)
Parity among 0 to 3 months breastfed^b^							
1 to 2 children (Ref)	144	25	Ref	---	9	Ref	---
≥ 3 children	108	16	1.16	(0.53, 2.56)	7	1.82	(0.57, 5.80)
Parity among ≥ 4 months breastfed^b^							
1 to 2 children (Ref)	358	52	Ref	---	14	Ref	---
≥ 3 children	352	44	0.99	(0.63, 1.55)	17	1.23	(0.57, 2.66)
Alcohol use							
Never (Ref)	709	110	Ref	---	39	Ref	---
Ever	880	129	0.98	0.73, 1.30	40	0.94	0.59, 1.50
Smoking history							
Never (Ref)	921	146	Ref	---	51	Ref	---
≤ 10	251	41	0.95	0.65, 1.40	7	0.51	0.23, 1.14
11 to 19	147	21	0.89	0.54, 1.47	8	1.17	0.54, 2.56
≥ 20	531	78	0.98	0.72, 1.34	26	1.05	0.63, 1.73
Hormone replacement therapy (postmenopausal only)							
Never (Ref)	314	50	Ref	---	24	Ref	---
Ever	943	112	0.83	0.57, 1.20	33	0.45	0.26, 0.79
Oral contraceptive use							
Never (Ref)	647	80	Ref	---	29	Ref	---
Ever	1160	202	0.97	0.72, 1.31	63	1.12	0.69, 1.83
BMI (kg/m^2^)^c^							
< 25 (Ref)	785	110	Ref	---	39	Ref	---
25 to 29	563	99	1.33	0.98, 1.81	31	1.21	0.74, 1.99
≥ 30	500	77	1.04	0.75, 1.45	23	1.03	0.59, 1.78
BMI (kg/m^2^) among premenopausal^b^							
< 25 (Ref)	223	39	Ref	---	7	Ref	---
25 to 29	99	28	1.82	1.03, 3.24	7	2.15	0.70, 6.58
≥ 30	81	23	1.97	1.03, 3.77	7	2.51	0.74, 8.51
BMI (kg/m^2^) among postmenopausal^b^							
< 25 (Ref)	480	59	Ref	---	25	Ref	---
25 to 29	422	60	1.08	0.73, 1.59	18	0.86	0.46, 1.61
≥ 30	372	43	0.76	0.49, 1.17	14	0.76	0.38, 1.51

^a ^Adjusted for age at diagnosis, race/ethnicity, and Pathways/LACE study origin except in models with age at diagnosis or race/ethnicity as main predictors.

^b ^*P *for interaction not statistically significant (*P *> 0.05) in any tumor subtype model.

^c ^BMI, body mass index one year pre-diagnosis (LACE) and around diagnosis (Pathways).

CI = confidence interval; HER2 = human epidermal growth receptor 2; OR = odds ratio.

For the subgroup analyses, among non-breastfeeding cases, parity of at least three children was associated with a statistically significant increased likelihood of having a triple-negative tumor (OR = 1.68; 95% CI = 1.00 to 2.81) and a Her2-overexpressing tumor (OR = 3.03; 95% CI = 1.27 to 7.23) compared with luminal A. In contrast, no differential associations of breastfeeding and parity were observed among luminal B cases. All *P *values for interaction of parity by breastfeeding were not statistically significant. As for the effect of BMI by menopausal status, premenopausal triple-negative and Her2-overexpressing cases were more likely to be overweight (triple negative: OR = 1.82, 95% CI = 1.03 to 3.24; Her2-overexpressing: OR = 2.15, 95% CI = 0.70 to 6.58) or obese (triple negative: OR = 1.97, 95% CI = 1.03 to 3.77; Her2-overexpressing: OR = 2.51, 95% CI = 0.74 to 8.51) at diagnosis, yet the effect measures for Her2-overexpressing tumors were not statistically significant. Among luminal A cases, these associations were not observed. All *P *values for interaction of BMI by menopausal status were not statistically significant.

## Discussion

In a pooled analysis of 2544 breast cancer cases using data from two prospective cohort studies housed within a large health maintenance organization, associations between breast cancer subtypes and various demographic, reproductive, and lifestyle factors were examined. In case-case analyses with the luminal A cases as the reference group, luminal B cases were more likely to be younger at diagnosis and were less likely to consume alcohol, use HRT, and OCs. Triple-negative cases tended to be younger at diagnosis and African American, and were more likely to be overweight and/or obese at diagnosis if premenopausal. Women with triple-negative tumors were also less likely to breastfeed for longer periods, and were more likely to not breastfeed if they had at least three children. Her2-overexpressing cases were more likely to be younger at diagnosis and Hispanic or Asian, and less likely to use HRT. We also found that these cases were more likely to be women with at least three children and no history of breastfeeding. These case-case observations suggest that heterogeneity in associations with traditional breast cancer risk factors exists by tumor subtype.

Several studies have assessed risk factor profiles of tumor subtypes, including the Carolina Breast Cancer Study (CBCS; n = 1424 *in situ *and invasive cases) [22], the Polish Breast Cancer Study (PBCS; 804 invasive cases) [27], and a pooled study of two Washington State (WS) case-control studies (n = 1023 invasive cases) [42,43] [see Additional data file 1]. The CBCS and PBCS were able to classify their triple-negative cases into basal-like and unclassified using CK5/6 and EGFR IHC expression data while the WS study did not do so. The CBCS performed case-case and case-control analyses while the PBCS and WS study conducted case-control analyses only. Although we were unable to further classify triple-negative cases into basal-like and unclassified, similar to the results of the CBCS (case-case analysis) and PBCS (case-control analysis) for basal-like cases, our triple-negative cases were more likely to be younger at diagnosis and African American. We also observed that premenopausal triple-negative cases tended to have higher BMI, which was in agreement with the basal-like cases in the CBCS but not the PBCS, the latter of which found no association. Interestingly, the WS study (case-control analysis) reported a suggestive increased risk of triple-negative tumors with increasing BMI among women currently using hormone therapy [42], yet we did not see any such association in our study. The WS study (case-control analysis) also reported that breastfeeding for at least six months was related to a reduced risk of triple-negative tumors [43]. Similarly, both the CBCS (case-case analysis) and our study found suggestive associations of shorter duration of breastfeeding (less than four months) with being more likely to have a triple-negative tumor. Furthermore, both studies observed a strong positive association for triple-negative cases (basal-like cases for CBCS) among women who had higher parity and never breastfed; the CBCS reported a case-only OR for parity of at least three children and no breastfeeding as 1.9 (95% CI = 1.1 to 3.4) compared with luminal A cases. The PBCS (case-control analysis) did not present data on the impact of breastfeeding on tumor subtypes.

As for luminal B and Her2-overexpressing cases, our results are in agreement with those of the CBCS that luminal B and Her2-overexpressing cases tended to be younger than luminal A cases. In contrast to the CBCS results, we observed that these cases were less likely to use HRT although luminal B cases were less likely to consume alcohol. No associations with these factors were observed in the PBCS, and the WS study did not examine luminal tumors separately by luminal A and luminal B subtype. We found that Her2-overexpressing cases were more likely to be Hispanic or Asian, but not African American, an observation which was not seen in any of the other studies. In fact, the CBCS, comprised of only whites and African Americans, reported that Her2-overexpressing cases were slightly more likely to be African American. Finally, we observed that Her2-overexpressing cases were more likely to be women who had at least three children and had not breastfed, an association not seen in the CBCS.

Although our results tend to be in agreement with those of other studies, limitations of our study should be discussed. Only case-case comparisons were conducted, and it must be emphasized that the associations reported here are all in reference to risk of having a luminal A tumor subtype and should not be extended to risk of having invasive breast cancer. Case-case analyses among tumor subtypes are a useful exploratory tool to examine etiologic heterogeneity between the subtypes [53]. As previously mentioned, we have no data on CK5/6 and EGFR tumor markers to further classify triple-negative tumors into basal-like and unclassified. However, with additional funding, we plan to conduct these additional IHC assays in triple negative cases. Also, as there were a limited number of Her2-overexpressing tumors (n = 94; 3.7%), results concerning this subtype should be interpreted with caution. Finally, although our large study population of 2544 women diagnosed with invasive breast cancer was more ethnically diverse (76.6% white, 6.1% African American, 7.8% Hispanic, 7.4% Asian, 2.1% other) than other studies that have examined breast cancer risk factors among tumor subtypes, unlike the CBCS, we were unable to further examine risk factors by white and African American race/ethnicity due to limited numbers. Our findings, especially those regarding Hispanic and Asian differences, should be replicated in other population-based studies.

## Conclusions

In summary, using a case-case analysis to assess the associations between traditional breast cancer risk factors and breast cancer subtypes (luminal A, luminal B, triple negative, and Her2-overexpressing), we observed significant heterogeneity of associations by tumor subtype. These varying associations by subtype lend further support to the growing evidence base that breast cancer is a heterogeneous disease defined by ER, PR, and Her2 expression with distinct etiologic pathways and prognoses. Future research should focus on refinement of tumor subtypes into more homogenous subgroups in order to best elucidate how risk factors may vary by subtype. Important modifiable factors that may be related to the development of specific tumor subtypes include obesity and possibly breastfeeding (triple negative) and alcohol consumption (luminal B), yet no clear modifiable risk factor profile was apparent for Her2-overexpressing subtypes due to a limited sample size. Given this information, public health programs aimed towards achieving a healthy weight and promoting breastfeeding might reduce the number of poor prognostic triple negative tumors among all breast cancer cases, especially the high-risk African American group.

## Abbreviations

BMI: body mass index; CBCS: Carolina Breast Cancer Study; CI: confidence interval; CK: cytokeratin; EGFR: epidermal growth factor receptor; ER: estrogen receptor; FISH: fluorescence *in situ *hybridization; Her2: human epidermal growth factor receptor 2; HRT: hormone replacement therapy; IHC: immunohistochemical; IRB: institutional review board; KPNC: Kaiser Permanente Northern California; LACE: Life After Cancer Epidemiology; OC: oral contraceptive; OR: odds ratio; PBCS: Polish Breast Cancer Study; PR: progesterone receptor; WS: Washington State.

## Competing interests

The authors declare that they have no competing interests.

## Authors' contributions

MLK contributed to study conception and design, and acquisition, analysis, and interpretation of data; and drafted and revised the manuscript critically for important intellectual content. LHK and BJC contributed to study conception and design, and acquisition, analysis, and interpretation of data; and revised the manuscript critically for important intellectual content. EW contributed to analysis and interpretation of data; and revised the manuscript critically for important intellectual content. BM contributed to study conception and design; and revised the manuscript critically for important intellectual content. SEK contributed to analysis and interpretation of data; and revised the manuscript critically for important intellectual content. RSF contributed to acquisition of data; and revised the manuscript critically for important intellectual content. MML contributed to analysis and interpretation of data; and revised the manuscript critically for important intellectual content. CBA contributed to analysis and interpretation of data; and revised the manuscript critically for important intellectual content. All authors read and approved the final manuscript.

## Supplementary Material

Additional file 1 A word file containing a table that lists previous studies that have examined associations between common breast cancer risk factors and breast cancer tumor subtypes. Click here for file

Additional file 2 A word file containing a table that lists the distribution of demographic, reproductive, and lifestyle risk factors in the LACE and Pathways studies individually and combined (n = 2544). Click here for file

Additional file 3 A word file containing a table that lists case-only odds ratios and 95% confidence intervals from logistic regression models of associations between breast cancer tumor subtypes and demographic, reproductive, and lifestyle risk factors, LACE study (n = 1821). Click here for file

Additional file 4 A word file containing a table that shows case-only odds ratios and 95% confidence intervals from logistic regression models of associations between breast cancer tumor subtypes and demographic, reproductive, and lifestyle risk factors, Pathways study (n = 723). Click here for file
