# Supplementary material for: Epidemiology of breast cancer subtypes in two prospective cohort studies of breast cancer survivors
Source: Breast Cancer Res. 2009 May 22;11(3):R31. doi: 10.1186/bcr2261 (PMC2716499; doi:10.1186/bcr2261)
Supplement: Additional file 1 — A word file containing a table that lists previous studies that have examined associations between common breast cancer risk factors and breast cancer tumor subtypes. [file bcr2261-S1.doc]

**Additional data file 1.** Previous studies that have examined associations between common breast cancer risk factors and breast cancer tumor subtypes

| **Author, Year, Location, Racial/Ethnic Composition** | **Analysis Approach** | **Sample Size**  **n (%)** | **Common Breast Cancer Risk Factors Examined^a^** |
| --- | --- | --- | --- |
| Yang 2007 [27]  Warsaw & Lodz, Poland  100% White | case-control analysis | Total = 804 invasive breast tumors  Luminal A = 552 (68.6)  Luminal B = 48 (6.0)  Triple Negative = 143 (17.8)  Basal-like = 95 (11.8)  Unclassified = 48 (6.0)  Her2+/ER- = 61 (7.6)  Total = 2,502 controls frequency matched to cases on city and age | age, education, age at menarche, age at first full-term birth, number of full-term births, menopausal status, age at menopause, BMI, oral HRT, family history, previous benign breast disease |
| Millikan 2008 [22]  24 counties, North Carolina, US  41% African American,  59% White | case-case and case-control analyses | Total = 1,424 *in situ* & invasive breast tumors  Luminal A = 796 (55.9)  Luminal B = 137 (9.6)  Triple Negative = 375 (26.3)  Basal-like = 225 (15.8)  Unclassified = 150 (10.5)  Her2+/ER- = 116 (8.2)  Total = 2,022 controls frequency matched to cases by age and race using randomized recruitment | age, race, menopausal status, family history, age at menarche, parity, age at first full-term pregnancy, lifetime duration lactation, alcohol use, smoking duration, oral contraceptive use, HRT, BMI, waist-hip ratio |
| Phipps 2008a [42]  3 counties, Washington State, US  92% non-Hispanic White | case-control analysis | Total = 1,124 invasive breast tumors  Luminal A & B = 1,008 (89.7)  Triple Negative = 77 (6.8)  Her2+/ER- = 39 (3.5)  Total = 1,447 controls frequency matched to cases by age | BMI (overall, no current HT use, current HT use), weight (overall, no current HT use, current HT use), height, lifetime maximum BMI and weight, BMI and weight at age 30 years, weight change |
| Phipps 2008b [43]  3 counties, Washington State, US  92% non-Hispanic White | case-control analysis | Total = 1,140 invasive breast tumors  Luminal A & B = 1,023 (89.7)  Triple Negative = 78 (6.9)  Her2+/ER- = 39 (3.4)  Total = 1,476 controls frequency matched to cases by age | age at menarche, parity, number of live births, age at first live birth, breastfeeding history, type of menopause, age at menopause, HT use |

^a^ HRT, hormone replacement therapy; BMI, body mass index; HT, hormone therapy
