# Supplementary material for: Epidemiology of breast cancer subtypes in two prospective cohort studies of breast cancer survivors
Source: Breast Cancer Res. 2009 May 22;11(3):R31. doi: 10.1186/bcr2261 (PMC2716499; doi:10.1186/bcr2261)
Supplement: Additional file 2 — A word file containing a table that lists the distribution of demographic, reproductive, and lifestyle risk factors in the LACE and Pathways studies individually and combined (n = 2544). [file bcr2261-S2.doc]

**Additional data file 2.** Distribution of demographic, reproductive, and lifestyle risk factors in the LACE and Pathways Studies individually and combined (n=2,544)

|  | **LACE**  **Total n=1,821**  **n (%)** | **Pathways**  **Total n=723**  **n (%)** | **Total**  **Total n=2,544**  **n (%)** | **p value^a^** |
| --- | --- | --- | --- | --- |
| Age at Diagnosis (years)  <50  50-64  ≥65  mean (±SD) | 427 (23.4)  821 (45.1)  573 (31.5)  58.6 (11.0) | 148 (20.5)  335 (46.3)  240 (33.2)  59.6 (12.2) | 575 (22.6)  1,156 (45.4)  813 (32.0)  58.9 (11.4) | 0.260 |
| Race/ethnicity  White  African American  Hispanic  Asian  Other | 1,430 (78.7)  106 (5.8)  128 (7.0)  120 (6.6)  33 (1.8) | 513 (71.2)  49 (6.8)  69 (9.6)  69 (9.6)  21 (2.9) | 1,943 (76.6)  155 (6.1)  197 (7.8)  189 (7.4)  54 (2.1) | 0.001 |
| Menopausal status  Postmenopausal  Premenopausal | 1,168 (74.3)  403 (25.7) | 511 (70.7)  212 (29.3) | 1,679 (73.2)  615 (26.8) | 0.065 |
| Family History  No  Yes | 1,453 (79.8)  367 (20.2) | 558 (77.2)  165 (22.8) | 2,011 (79.1)  532 (20.9) | 0.137 |
| Parity  Nulliparous  1-2 children  ≥3 children | 311 (17.1)  816 (44.9)  692 (38.0) | 140 (19.4)  350 (48.4)  233 (32.2) | 451 (17.7)  1,166 (45.9)  925 (36.4) | 0.021 |
| Age at first full-term pregnancy (years)  Nulliparous  <26  ≥26 | 311 (17.1)  999 (55.0)  507 (27.9) | 140 (19.4)  345 (47.9)  236 (32.7) | 451 (17.8)  1,344 (53.0)  743 (29.3) | 0.005 |
| Lifetime duration of lactation  Never  0-3 months  ≥4 months | 893 (50.0)  249 (13.9)  645 (36.1) | 305 (42.5)  115 (16.0)  297 (41.4) | 1,198 (47.8)  364 (14.5)  942 (37.6) | 0.003 |
| Alcohol use  Never  Ever | 678 (44.8)  835 (55.2) | 311 (48.3)  333 (51.7) | 989 (45.9)  1,168 (54.1) | 0.138 |
| Smoking duration  Never  ≤10 yrs  11-19 yrs  ≥20 yrs | 913 (50.6)  247 (13.7)  128 (7.1)  515 (28.6) | 371 (51.4)  91 (12.6)  71 (9.8)  189 (26.2) | 1,284 (50.9)  338 (13.4)  199 (7.9)  704 (27.9) | 0.091 |
| HRT (postmenopausal women only)^b^  Never  Ever | 276 (24.1)  867 (75.9) | 169 (33.5)  336 (66.5) | 445 (27.0)  1203 (73.0) | <0.0001 |
| Oral contraceptive use  Never  Ever | 673 (38.4)  1,081 (61.6) | 194 (27.2)  518 (72.8) | 867 (35.2)  1,599 (64.8) | <0.0001 |
| BMI (kg/m^2^)^b^  <25  25-29  ≥30 | 822 (45.7)  546 (30.4)  431 (24.0) | 248 (34.3)  229 (31.7)  246 (34.0) | 1,070 (42.4)  775 (30.7)  677 (26.8) | <0.0001 |
| Tumor subtype  Luminal A (ER+ and/or PR+, Her2–)  Luminal B (ER+ and/or PR+, Her2+)  Triple Negative (ER–, PR–, Her2–)  Her2-overexpressing (ER–, PR–, Her2+) | 1329 (73.0)  225 (12.4)  199 (10.9)  68 (3.7) | 539 (74.6)  69 (9.5)  89 (12.3)  26 (3.6) | 1868 (73.4)  294 (11.6)  288 (11.3)  94 (3.7) | 0.202 |

^a^ From Pearson chi-square test across study origin

^b^ HRT, hormone replacement therapy; BMI, body mass index one year pre-diagnosis (LACE) and around diagnosis (Pathways)
