# Supplementary material for: Epidemiology of breast cancer subtypes in two prospective cohort studies of breast cancer survivors
Source: Breast Cancer Res. 2009 May 22;11(3):R31. doi: 10.1186/bcr2261 (PMC2716499; doi:10.1186/bcr2261)
Supplement: Additional file 3 — A word file containing a table that lists case-only odds ratios and 95% confidence intervals from logistic regression models of associations between breast cancer tumor subtypes and demographic, reproductive, and lifestyle risk factors, LACE study (n = 1821). [file bcr2261-S3.doc]

**Additional data file 3.** Case-only odds ratios and 95% confidence intervals from logistic regression models^a^ of associations between breast cancer tumor subtypes and demographic, reproductive, and lifestyle risk factors, LACE Study (n=1,821)

|  | **Luminal A**  **(comparison)** | **Luminal B^a^** | | | **Triple Negative^a^** | | | **HER2-overexpressing^a^** | | |
| --- | --- | --- | --- | --- | --- | --- | --- | --- | --- | --- |
|  | **n** | **n** | **OR** | **95% CI** | **n** | **OR** | **95% CI** | **n** | **OR** | **95% CI** |
| Age at diagnosis (years)  ≥65 (Ref)  50-64  <50 | 444  621  260 | 66  90  69 | Ref  0.97  1.71 | ---  0.69, 1.36  1.18, 2.49 | 43  74  82 | Ref  1.18  3.23 | ---  0.79, 1.75  2.15, 4.84 | 18  35  15 | Ref  1.38  1.36 | ---  0.77, 2.47  0.67, 2.76 |
| test for trend |  |  |  | p=0.001 |  |  | p≤0.0001 |  |  | p=0.35 |
| Race/ethnicity  White (Ref)  African American  Hispanic  Asian  Other | 1065  65  89  84  22 | 166  11  20  23  5 | Ref  1.00  1.35  1.60  1.38 | ---  0.52, 1.95  0.81, 2.27  0.97, 2.62  0.51, 3.71 | 149  27  13  7  3 | Ref  2.68  0.86  0.52  0.87 | ---  1.64, 4.38  0.46, 1.60  0.23, 1.15  0.25, 2.99 | 50  3  6  6  3 | Ref  0.95  1.39  1.48  2.81 | ---  0.29, 3.15  0.58, 3.33  0.62, 3.56  0.81, 9.73 |
| Menopausal status  Postmenopausal (Ref)  Premenopausal | 895  256 | 129  70 | Ref  1.52 | ---  0.92, 2.52 | 99  66 | Ref  1.08 | ---  0.64, 1.84 | 43  10 | Ref  0.62 | ---  0.23, 1.63 |
| Family History  No (Ref)  Yes | 1043  282 | 189  36 | Ref  0.73 | ---  0.50, 1.07 | 158  41 | Ref  0.99 | ---  0.68, 1.44 | 60  8 | Ref  0.50 | ---  0.24, 1.06 |
| Age at first full-term pregnancy (years)  Nulliparous (Ref)  <26  ≥26 | 226  710  387 | 37  128  60 | Ref  1.30  1.01 | ---  0.87, 1.96  0.64, 1.58 | 38  115  46 | Ref  1.23  0.86 | ---  0.82, 1.87  0.54, 1.38 | 10  45  13 | Ref  1.55  0.76 | ---  0.76, 3.18  0.33, 1.77 |
| Parity  Nulliparous (Ref)  1-2 children  ≥3 children | 226  586  513 | 37  109  79 | Ref  1.21  1.13 | ---  0.81, 1.83  0.73, 1.75 | 38  93  68 | Ref  1.07  1.11 | ---  0.70, 1.62  0.71, 1.75 | 10  27  31 | Ref  1.04  1.53 | ---  0.50, 2.21  0.72, 3.25 |
| Lifetime duration of breastfeeding  Never (Ref)  0-3 months  ≥4 months | 637  177  489 | 108  36  72 | Ref  1.24  0.81 | ---  0.82, 1.88  0.59, 1.12 | 110  27  61 | Ref  0.96  0.67 | ---  0.61, 1.54  0.48, 0.95 | 37  8  22 | Ref  0.78  0.75 | ---  0.36, 1.71  0.43, 1.29 |
| Alcohol use  Never (Ref)  Ever | 481  624 | 97  90 | Ref  0.72 | ---  0.52, 1.00 | 71  91 | Ref  0.97 | ---  0.69, 1.38 | 29  30 | Ref  0.83 | ---  0.48, 1.42 |
| Smoking history  Never (Ref)  ≤10  11-19  ≥20 | 645  185  96  386 | 126  29  14  55 | Ref  0.80  0.79  0.81 | ---  0.52, 1.25  0.44, 1.44  0.57, 1.16 | 107  28  12  50 | Ref  0.82  0.75  0.87 | ---  0.52, 1.30  0.39, 1.43  0.60, 1.26 | 34  5  5  23 | Ref  0.51  1.04  1.22 | ---  0.19, 1.33  0.39, 2.73  0.69, 2.13 |
| HRT (postmenopausal only) ^b^  Never (Ref)  Ever | 193  682 | 40  85 | Ref  0.58 | ---  0.38, 0.87 | 27  71 | Ref  0.72 | ---  0.45, 1.18 | 15  28 | Ref  0.53 | ---  0.27, 1.03 |
| Oral contraceptive use  Never (Ref)  Ever | 501  778 | 90  123 | Ref  0.72 | ---  0.52, 0.99 | 61  132 | Ref  0.92 | ---  0.65, 1.32 | 20  47 | Ref  1.50 | ---  0.84, 2.69 |
| BMI (kg/m^2^) ^b^  <25 (Ref)  25-29  ≥30 | 600  395  315 | 113  59  49 | Ref  0.89  0.93 | ---  0.63, 1.26  0.64, 1.35 | 77  69  51 | Ref  1.59  1.35 | ---  1.10, 2.28  0.90, 2.02 | 30  22  16 | Ref  1.20  1.09 | ---  0.67, 2.14  0.58, 2.07 |

^a^ Adjusted for age at diagnosis and race/ethnicity except in models with age at diagnosis or race/ethnicity as main predictors

^b^ HRT, hormone replacement therapy; BMI, body mass index one year pre-diagnosis
