# Supplementary material for: Epidemiology of breast cancer subtypes in two prospective cohort studies of breast cancer survivors
Source: Breast Cancer Res. 2009 May 22;11(3):R31. doi: 10.1186/bcr2261 (PMC2716499; doi:10.1186/bcr2261)
Supplement: Additional file 4 — A word file containing a table that shows case-only odds ratios and 95% confidence intervals from logistic regression models of associations between breast cancer tumor subtypes and demographic, reproductive, and lifestyle risk factors, Pathways study (n = 723). [file bcr2261-S4.doc]

**Additional data file 4.** Case-only odds ratios and 95% confidence intervals from logistic regression models^a^ of associations between breast cancer tumor subtypes and demographic, reproductive, and lifestyle risk factors, Pathways Study (n=723)

|  | **Luminal A**  **(comparison)** | **Luminal B^a^** | | | **Triple Negative^a^** | | | **HER2-overexpressing^a^** | | |
| --- | --- | --- | --- | --- | --- | --- | --- | --- | --- | --- |
|  | **n** | **n** | **OR** | **95% CI** | **n** | **OR** | **95% CI** | **n** | **OR** | **95% CI** |
| Age at diagnosis (years)  ≥65 (Ref)  50-64  <50 | 193  250  95 | 16  34  19 | Ref  1.66  2.37 | ---  0.88, 3.10  1.15, 4.89 | 25  41  23 | Ref  1.17  1.89 | ---  0.68, 2.01  1.00, 3.58 | 5  10  10 | Ref  1.40  2.83 | ---  0.46, 4.26  0.91, 8.82 |
| test for trend (p-value) |  |  |  | p=0.04 |  |  | p=0.03 |  |  | p=0.03 |
| Race/ethnicity  White (Ref)  African American  Hispanic  Asian  Other | 399  27  46  51  15 | 49  3  8  7  2 | Ref  0.86  1.22  0.93  1.08 | ---  0.25, 2.94  0.53, 2.78  0.39, 2.21  0.24, 4.87 | 55  17  8  5  4 | Ref  4.33  1.12  0.60  1.94 | ---  2.21, 8.49  0.50, 2.53  0.23, 1.59  0.62, 6.09 | 10  2  7  6  0 | Ref  2.61  4.84  3.49  --- | ---  0.54, 12.64  1.70, 13.78  1.18, 10.33  --- |
| Menopausal status  Postmenopausal (Ref)  Premenopausal | 388  150 | 44  25 | Ref  0.95 | ---  0.45, 1.99 | 64  25 | Ref  0.52 | ---  0.26, 1.04 | 14  11 | Ref  0.70 | ---  0.23, 2.16 |
| Family History  No (Ref)  Yes | 410  128 | 56  13 | Ref  0.77 | ---  0.40, 1.45 | 71  18 | Ref  0.85 | ---  0.48, 1.50 | 20  5 | Ref  1.09 | ---  0.39, 3.07 |
| Age at first full-term pregnancy (years)  Nulliparous (Ref)  <26  ≥26 | 109  249  180 | 14  30  25 | Ref  1.08  1.10 | ---  0.53, 2.18  0.55, 2.23 | 14  48  26 | Ref  1.34  1.11 | ---  0.67, 2.65  0.55, 2.24 | 3  16  5 | Ref  4.04  1.14 | ---  1.01, 16.07  0.25, 5.12 |
| Parity  Nulliparous (Ref)  1-2 children  ≥3 children | 109  251  178 | 14  41  14 | Ref  1.31  0.69 | ---  0.68, 2.53  0.31, 1.55 | 14  44  31 | Ref  1.20  1.33 | ---  0.62, 2.33  0.65, 2.73 | 3  13  9 | Ref  2.02  2.68 | ---  0.54, 7.58  0.64, 11.19 |
| Lifetime duration of breastfeeding  Never (Ref)  0-3 months  ≥4 months | 234  78  222 | 27  14  28 | Ref  1.57  1.01 | ---  0.78, 3.16  0.57, 1.78 | 38  14  36 | Ref  1.22  1.05 | ---  0.61, 2.41  0.63, 1.75 | 6  8  10 | Ref  3.82  1.67 | ---  1.24, 11.81  0.58, 4.80 |
| Alcohol use  Never (Ref)  Ever | 228  256 | 34  29 | Ref  0.79 | ---  0.45, 1.36 | 39  38 | Ref  0.94 | ---  0.57, 1.56 | 10  10 | Ref  1.46 | ---  0.55, 3.90 |
| Smoking history  Never (Ref)  ≤10  11-19  ≥20 | 276  66  51  145 | 37  10  8  13 | Ref  1.09  1.32  0.73 | ---  0.51, 2.33  0.57, 3.06  0.37, 1.45 | 39  13  9  28 | Ref  1.40  1.24  1.28 | ---  0.69, 2.81  0.55, 2.80  0.73, 2.22 | 17  2  3  3 | Ref  0.61  1.98  0.51 | ---  0.13, 2.81  0.51, 7.73  0.14, 1.87 |
| HRT (postmenopausal only) ^b^  Never (Ref)  Ever | 121  261 | 15  29 | Ref  0.97 | ---  0.49, 1.93 | 23  41 | Ref  1.00 | ---  0.55, 1.81 | 9  5 | Ref  0.30 | ---  0.10, 0.96 |
| Oral contraceptive use  Never (Ref)  Ever | 146  382 | 20  49 | Ref  0.76 | ---  0.42, 1.38 | 19  70 | Ref  1.03 | ---  0.57, 1.86 | 9  16 | Ref  0.60 | ---  0.23, 1.52 |
| BMI (kg/m^2^) ^b^  <25 (Ref)  25-29  ≥30 | 177  174  187 | 20  21  28 | Ref  1.10  1.43 | ---  0.57, 2.12  0.76, 2.68 | 33  30  26 | Ref  0.91  0.60 | ---  0.52, 1.59  0.33, 1.08 | 9  9  7 | Ref  1.07  0.84 | ---  0.40, 2.88  0.29, 2.50 |

^a^ Adjusted for age at diagnosis and race/ethnicity except in models with age at diagnosis or race/ethnicity as main predictors

^b^ HRT, hormone replacement therapy; BMI, body mass index around diagnosis
